# Management of Spigelian hernia caused by necrobiotic fibroma of the uterus in a pregnant woman^☆^

**DOI:** 10.1016/j.ijscr.2013.10.010

**Published:** 2013-10-24

**Authors:** Radwan Kassir, Enrico Tarantino, Robert Lacheze, Amine Brek, Aurelie Di Bartolomeo, Olivier Tiffet

**Affiliations:** a Department of Digestive Surgery, CHU Hospital, Jean Monnet University, Saint Etienne, France; b Department of General Surgery, CHU Hospital, Jean Monnet University, Saint Etienne, France; c Department of Radiology, CHU Hospital, Jean Monnet University, Saint Etienne, France; d Department of Gynecology and Obstetrics, CHU Hospital, Jean Monnet University, Saint Etienne, France

**Keywords:** Spigelian hernia, Necrobiotic fibroma, Pregnant woman, Magnetic Resonance Imaging, Surgery

## Abstract

**INTRODUCTION:**

Spigelian hernias are a rare type of hernia through the Spigelian aponeurosis. Spigelian hernias are very uncommon and constitute only 0.12% of all abdominal wall hernias. These hernias are located in the aponeurosis of the internal oblique muscle and transverse abdominal muscle.

**PRESENTATION OF CASE:**

A 30-year-old woman at 28 weeks’ gestation was admitted to the obstetrics department due to pain and swelling in the anterior abdominal right region. On inspection, we suspected either a lipoma, a spontaneous hematoma, a tumor of the abdominal wall, or a Spigelian hernia. A Doppler USG and abdominal and pelvic Magnetic Resonance Imaging revealed necrobiotic fibroma of the uterus in Spigelian hernia. The patient was started on dual analgesic and corticotherapy. Overall, the patient improved one week after the acute episode and had no further pain during her gynecologic follow-up.

**DISCUSSION:**

We have reported a first case of Spigelian hernia that was complicated by uterine fibroid. The clinical presentation varies, depending on the contents of the hernial sac and the degree of herniation. MRI is the preferred method for accurately identifying masses of the abdominal wall. Our treatment options were based on the extent of the acute-phase reaction and the venous thrombosis.

**CONCLUSION:**

It is important to differentiate this rare Spigelian hernia from other hernias as the treatment for this hernia is medical rather than surgical. Before the final choice of treatment is made, digestive surgeons should bear this rare hernia in mind.

## 1 Introduction

Spigelian hernias are a rare type of hernia through the Spigelian aponeurosis. Spigelian hernias occur due to a slit-like defect in the anterior abdominal wall adjacent to the semilunar line. Diagnosis of Spigelian hernias is difficult. In the absence of incarceration or strangulation. Smooth muscle tumors are the most common type of uterine neoplasm. We will present below a first case of Spigelian hernia caused by necrobiotic fibroma of the uterus in a pregnant woman.

## 2 Presentation of case

A 30-year-old woman (gravida 1 para 0) at 28 weeks’ gestation was admitted to the department of gynecology and obstetrics due to pain and swelling in the anterior abdominal right region. This mass had suddenly appeared two months before. Her pregnancy was progressing well (body mass index of 16 kg/m^2^) and she had no personal or family medical or surgical history. She had no history of medical drug use.

**Fig. 1:**
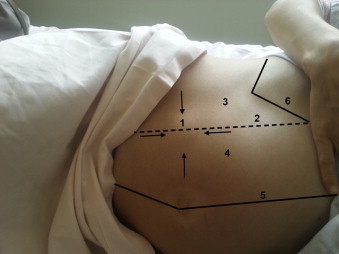
Image of the patient's abdomen with a Spigelian hernia. (1) Spigelian hernia. (2) Spigelian aponeurosis. (3) The transversus abdominis. (4) Rectus abdominis muscle. (5) Linea alba. (6) Costal cartilage.

On admission, she had no fever, vomiting or transit disorder. On inspection, we suspected a lipoma, a spontaneous hematoma, a tumor of the abdominal wall or a Spigelian hernia. Physical examination revealed a Spigelian hernia that was rather painful because the mass had developed on the linea arcuata (Fig. 1). This mass was not reducible. Her laboratory findings were within normal. The fetus was active and fluid was normal with fetal parameters corresponding to a gestational age of 28 weeks.

**Fig. 2:**
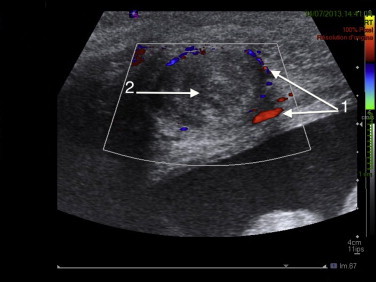
Doppler USG of the anterior abdominal area. (1) Circumferential vascularity. (2) Absence of flow.

**Fig. 3:**
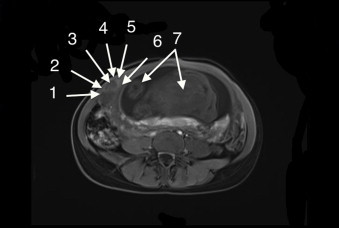
Abdominal Magnetic Resonance Imaging-T1 was performed and revealed uterine fibroid in Spigelian hernia (arrow). (1) Transverse abdominis. (2) Internal oblique. (3) Spigelian hernia. (4) Linea Semilunaris. (5) Rectus abdominis. (6) Uterus. (7) Fetus.

**Fig. 4:**
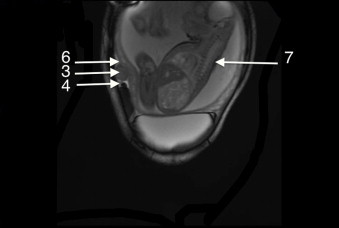
Abdominal Magnetic Resonance Imaging-T2 was performed and revealed uterine fibroid in Spigelian hernia (arrow). (3) Spigelian hernia. (4) Linea Semilunaris. (6) Uterus. (7) Fetus.

Ultrasound assessment of the anterior abdominal area revealed a mass on the aponeurotic layer between the rectus abdominis muscle medially and the semilunar line laterally. The mass appeared well-defined, solid, with a whorled appearance and of similar echogenicity to the myometrium. The Doppler USG showed circumferential vascularity and absence of flow (Fig. 2). Typically, it has a fibroid that had undergone torsion. Abdominal and pelvic Magnetic Resonance Imaging was performed and showed a mass isointense to the myometrium on T1W images (Fig. 3) and low-signal intensity compared to the myometrium on T2W images (Fig. 4). Abdominal and pelvic Magnetic Resonance Imaging revealed necrobiotic fibroma of the uterus in Spigelian hernia.

The patient was started on dual analgesic and corticotherapy. Overall, the patient improved one week after the acute episode and had no further pain during her gynecologic follow up.

## 3 Discussion

We have reported a first case of Spigelian hernia that was complicated by uterine fibroid. Spigelian hernias are very uncommon and constitute only 0.12% of all abdominal wall hernias.^1^ First described in 1764, the hernia of the semilunar line is called a Spigelian hernia.^2^ Also known as spontaneous lateral ventral hernias, a Spigelian hernia is the protrusion of organ(s) due to defect in the Spigelian aponeurosis. Spigelian hernias are located in the aponeurosis of the internal oblique muscle and transverse abdominal muscle. Spigelian hernias can be acquired or congenital.^3^ The Spigelian aponeurosis is widest between 0 and 6 cm cranial to the interspinous plane and 85–90% of the hernias occur within this “Spigelian hernia” belt.^4^

The clinical presentation varies, depending on the contents of the hernial sac and the degree of herniation. Because of its varied presentation, clinical examination is often inconclusive. In this case, the diagnosis was apparent because the patient had a palpable lump along the Spigelian aponeurosis.

Only 50% of cases are diagnosed preoperatively.^5^ Ultrasound is recommended as first line imaging investigation. The differential diagnosis includes appendicitis and appendiceal abscess, a tumor of the abdominal wall, a spontaneous hematoma of the rectus sheath or even acute diverticulitis.^6^ If after radiological investigation the diagnosis is uncertain, diagnostic laparoscopy may be performed. Care must be taken not to create iatrogenic Spigelian hernias when using laparoscopy trocars or classic drains of the Spigelian aponeurosis.^7^

The clinical was resolved entirely with analgesics and corticotherapy. Our treatment options were based on the extent of the acute-phase reaction and the venous thrombosis.

Smooth muscle tumors are the most common type of uterine neoplasm. The vast majority of such tumors are benign leiomyomas. Leiomyomas are by far the most frequent mesenchymal uterine neoplasms. The leiomyoma, by far the most common of all the neoplasms, is generally hormone sensitive, with rates of growth semi-quantitatively related to estrogen and progesterone receptor levels. Ischemic or infarct type necrosis is not considered a criterion for malignancy. The diagnosis of fibroids on USG is usually reasonably straightforward. When there is doubt about the origin of a mass at USG, further evaluation by means of MRI should be performed. MRI is the preferred method for accurately identifying masses of the abdominal wall. The risk of strangulation of uterine fibroid and necrobiotic is higher because of the sharp fascial margin around the defect.

Before the final choice of treatment is made, digestive surgeons should bear this rare hernia of uterine fibroid in mind for the differential diagnosis of an intestinal incarceration. It is important to differentiate this rare Spigelian hernia from other hernias, as the treatment is medical rather than surgical. Indeed, this mass has a remarkably favorable prognosis.

## Conflict of interest statement

None declared.

## Funding

None.

## Ethical approval

Written informed consent was obtained from the patient for publication of this case report and accompanying images. A copy of the written consent is available for review by the Editor-in-Chief of this journal on request.

## Author contributions

Radwan Kassir and Enrico Tarantino contributed in writing the paper. Robert Lachez and Aurelie Di Bartolomeo collected the data. Amine Brek and Olivier Tiffet reviewed and revised the paper.
